# When Money Is Not Enough: Awareness, Success, and Variability in Motor Learning

**DOI:** 10.1371/journal.pone.0086580

**Published:** 2014-01-28

**Authors:** Harry Manley, Peter Dayan, Jörn Diedrichsen

**Affiliations:** 1 Institute of Cognitive Neuroscience, University College London, London, United Kingdom; 2 Gatsby Computational Neuroscience Unit, University College London, London, United Kingdom; 3 Institute for the Psychology of Elite Performance, Bangor University, Bangor, United Kingdom; VU University Amsterdam, Netherlands

## Abstract

When performing a skill such as throwing a dart, many different combinations of joint motions suffice to hit the target. The motor system adapts rapidly to reduce bias in the desired outcome (i.e., the first-order moment of the error); however, the essence of skill is to produce movements with less variability (i.e., to reduce the second-order moment). It is easy to see how feedback about success or failure could sculpt performance to achieve this aim. However, it is unclear whether the dimensions responsible for success or failure need to be known explicitly by the subjects, or whether learning can proceed without explicit awareness of the movement parameters that need to change. Here, we designed a redundant, two-dimensional reaching task in which we could selectively manipulate task success and the variability of action outcomes, whilst also manipulating awareness of the dimension along which performance could be improved. Variability was manipulated either by amplifying natural errors, leaving the correlation between the executed movement and the visual feedback intact, or by adding extrinsic noise, decorrelating movement and feedback. We found that explicit, binary, feedback about success or failure was only sufficient for learning when participants were aware of the dimension along which motor behavior had to change. Without such awareness, learning was only present when extrinsic noise was added to the feedback, but not when task success or variability was manipulated in isolation; learning was also much slower. Our results highlight the importance of conscious awareness of the relevant dimension during motor learning, and suggest that higher-order moments of outcome signals are likely to play a significant role in skill learning in complex tasks.

## Introduction

**Figure 1:**
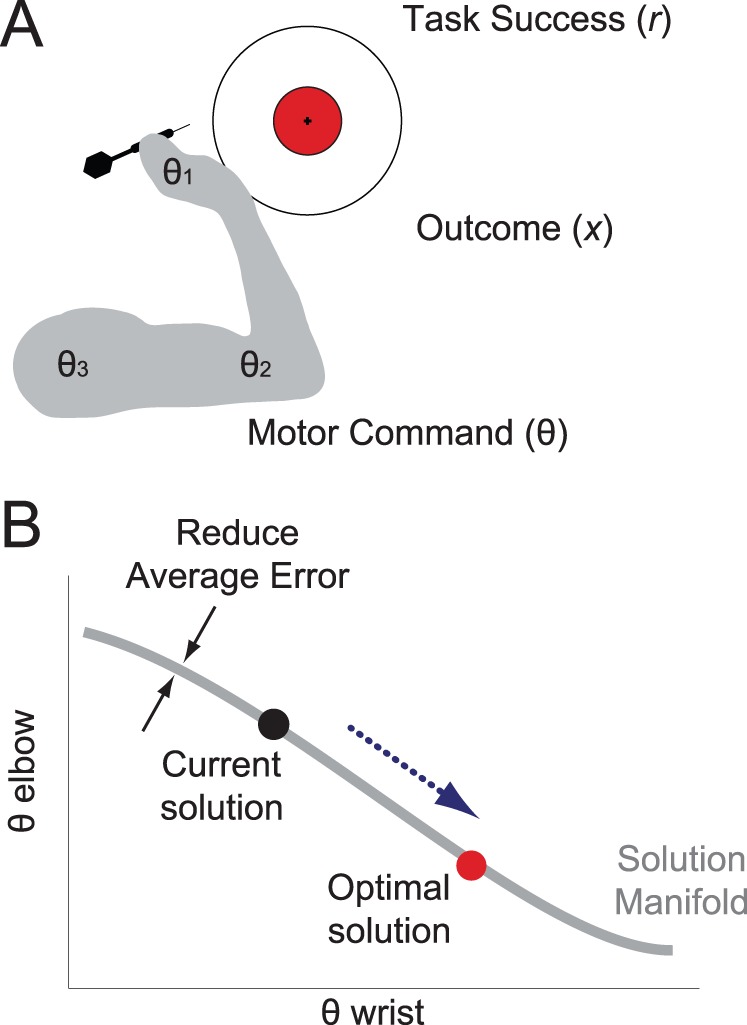
The problem of finding an optimal solution in a redundant system. (A) When throwing a dart, the motor commands are characterized by a high-dimensional parameter vector (θ). The exact setting of control parameters then determines a low-dimensional outcome *x*, the location where the dart lands, which in turn determines task success (*r*, the number of points obtained). In order to improve performance, the motor system must use the reward signal *r*, or an appropriate statistic on the outcome (for example the variability of *x*) to change the appropriate components in θ. (B) Example with two components of θ and a one-dimensional *x*. Many combinations of θ_ elbow_ and θ_ wrist_ result in zero error on average, forming a lower-dimensional subspace called the solution manifold (gray line). Error-based learning can keep the motor system on this solution manifold, but does not provide a mechanism by which to find the best solution (red circle) on the solution manifold.

How do people learn complex motor skills such as playing a musical instrument or downhill skiing? One special challenge in learning new motor behaviors is the redundancy inherent in many tasks and in human biomechanics. Take, for example, the game of darts. The outcome variable that ultimately matters (the location where the dart hits the board) is determined by a large number of variables the motor system must control (here denoted as the movement parameter vector θ), including the posture of the trunk, the velocity and position of the shoulder and elbow joints, the orientation of the wrist, and the exact timing of the dart’s release (Fig. 1a) [1]–[3]. In a simplified 2-dimensional example (Fig. 1b), the vertical position of the dart (the outcome variable *x*) may depend on two parameters that determine this movement, for example the angle of the elbow (θ_elbow_) and the angle of the wrist (θ_wrist_) at the moment of release. Multiple combinations of these parameters can achieve zero error *on average*. Such solutions form a lower-dimensional subspace in the high-dimensional parameter space called the uncontrolled or *solution manifold* [4], [5].

When learning this skill, one important learning mechanism is (first-order) error-based learning [6]–[8]. This mechanism can be demonstrated by asking participants to wear prism glasses that shift the visual world to one side. Since the motor system assumes the calibration between movements and visual outcomes to be normal, the dart will miss the dartboard in the direction of the shift on the very first throw. Based on this error, the motor system adapts the next motor command to make the dart strike a bit closer to the board [9]–[11]. Thus first-order error-based learning leads to fast improvements by bringing the system back to the solution manifold (Fig. 1b).

However, not all solutions on the manifold are equally good. Some may demand less effort; others may reduce the variability of the final outcome, either because in this region of the parameter space the motor noise is lower, or because in this region, variability in θ does not cause large variability in *x* [12]. Because the average (signed) performance error is zero throughout the solution manifold, first-order error-based learning cannot be instrumental in finding the most reliably successful solution. For this, a straightforward strategy for the motor system is to explore different solutions, and find one that leads to a lower variance or a higher success rate [12], [13]. Here we ask which teaching signals and learning algorithms underpin this capacity.

The most obvious teaching signal is the explicit success of the task at hand. This is suitable for all forms of direct and indirect reinforcement-learning rules [14]–[16]. In darts, for instance, success is determined by the points obtained for each throw. Because the mapping between the movement outcome and task success can be directly manipulated, the role of explicit task success can be easily tested. Indeed, two recent studies [17], [18] used tasks in which the reward provided for a reaching movement depended on the reach direction. By shifting the rewarded zone to one side, both studies’ authors systematically induced changes in participants’ reach directions. The second study [18] also showed that these changes were qualitatively different from learning induced by error-based learning from visual feedback. Based on these studies, it appears that arbitrary manipulation of task success feedback can drive learning in the motor system.

However, in both studies, the reward probability varied along a single dimension of control that participants knew would matter for task achievement, namely the reach direction. Thus, when failing to achieve task success, participants actively explored the range of possible reach directions until they found the target zone again. In many real-life motor tasks, however, people are often unaware of the dimension(s) they must vary in order to improve performance. Reinforcement learning in complex tasks therefore constitutes a difficult estimation problem. For example, in dart throwing, the learner is not certain about whether to change wrist or elbow angle, whether to vary throwing speed, or whether to change the postural configuration of the trunk. Indeed, a central role for a coach is to reduce this uncertainty by making these critical variables apparent. Therefore, the first question we addressed in this paper is whether explicit information about task success alone can guide learning, or whether awareness of the relevant control parameter is necessary for learning to occur.

Secondly, we asked whether other signals, apart from explicit task success, play a role in the learning process: the motor system receives more detailed information about the motor outcome *x* than the relatively sparse signal of task success (hit or no hit). Whereas error-based learning uses the first moment (i.e., the average) of *x*, the system could also use information about higher order moments (i.e., forms of variability) of *x* for learning.

Many normative theories of motor control indeed hold that the motor system strives to find solutions that minimize outcome variability [19]–[22]. Under most circumstances, reduction in variability also leads to increased task success, making these theories difficult to distinguish from models that posit that the motor system learns based on explicit rewards. Our second goal was to test whether the observed outcome variability plays a role as a signal for motor learning, independent of task success.

Note, however, that variability in the observed outcome is not unitary. It can arise from intrinsic sources, such as noise in central planning processes [23], or from extrinsic sources, such as externally imposed perturbations. It is conceivable that the motor system can distinguish between these two sources, by relating information about the executed movement (using efference copy from the outgoing motor command) with the observed movement outcome. A high correlation would indicate an intrinsic source, which at least might be controllable. By contrast, a lack of correlation would indicate an extrinsic source, which is likely to be uncontrollable. This characteristic difference in controllability suggests that extrinsic and intrinsic sources of noise may be treated differently by the motor system, and we therefore tested whether they differed in their ability to induce learning. By visually magnifying the movement error made by the participants, one can increase variability, whilst leaving the correlation between the executed and observed movements intact. By adding random noise to the feedback instead, the variability can be increased by the same amount, whilst simultaneously decorrelating movement and error feedback.

**Figure 2:**
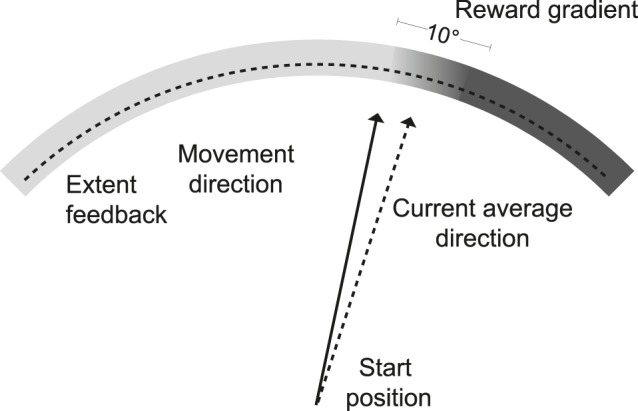
Task design. Participants had to execute a reaching movement from the start position towards an arced target. The explicit task goal was to hit the target in terms of movement extent, using any movement direction from the start position. Visual feedback was provided at movement end in the form of an arced line that indicated reach extent only. The program continuously estimated the average movement direction based on recent movements (dashed line) and centered a gradient for the 10° around this direction. Based on this gradient (which was not visually shown to the participant), a movement (solid line) was rewarded with a probability between 0.2 (lighter) and 0.8 (darker). Learning should therefore change movement direction towards the more highly rewarded region.

To investigate the role of awareness and various teaching signals in learning along the solution manifold, we therefore needed a task involving redundancy in which we could manipulate noise and success independently. We used a redundant reaching task [24], in which participants were instructed to hit an arc-shaped target using any reach direction from the origin (Fig. 2). The task-relevant outcome *x* was therefore the reach extent, indicated by an arc-shaped cursor. Reach direction was not *directly* important for task success. Therefore the solution manifold for this task encompassed all reach directions, as at any reach direction a zero-error movement could be achieved. However, different reach directions (i.e., positions on the solution manifold) were made to differ in how, and how well, reach extent could be controlled and therefore how successful the movement would be on average. We manipulated the three learning signals, task success, variability in movement amplitude, and the correlation between the action and the outcome, to determine their separate and joint effects on learning along the solution manifold.

## Methods

### Task Procedure

Participants made reaching movements in the horizontal plane, while holding onto a robotic arm. Visual feedback was provided at the end of the movement with an arced cursor that indicated reach extent but not reach direction (Fig. 2). The goal of the task was to land the cursor in the middle of the target, displayed with radial width 0.75 cm, making reach extent the task-critical outcome variable (x), while reach direction was (apparently) not directly important. Reaches that terminated in the rewarded zone (usually the target) within 700 ms resulted in participants receiving a small monetary reward (1 pence), indicated by a pleasing sound and a visually animated explosion of the target. The current score was continuously displayed on the screen using a point counter, and participants were paid at the end of the experiment based on their final score. Unbeknownst to all but five participants, we placed a gradient along the solution manifold (the extent of the elongated target) that made it easier to score points on one side of the target, and/or that manipulated the visual feedback about the reach amplitude. To ensure that participants would experience the whole gradient, we varied the probability of success over a small range (10°) around an estimate of the current mean movement direction of the participant (up to ±25° from the center). The gradient was divided into ten equally sized, distinct regions. Movements to the left or right of this 10° window experienced the same manipulation of the feedback as the movements to the endpoints of the window. Five participants were explicitly informed that reach direction could affect the outcome (see below under the awareness manipulation).

**Figure 3:**
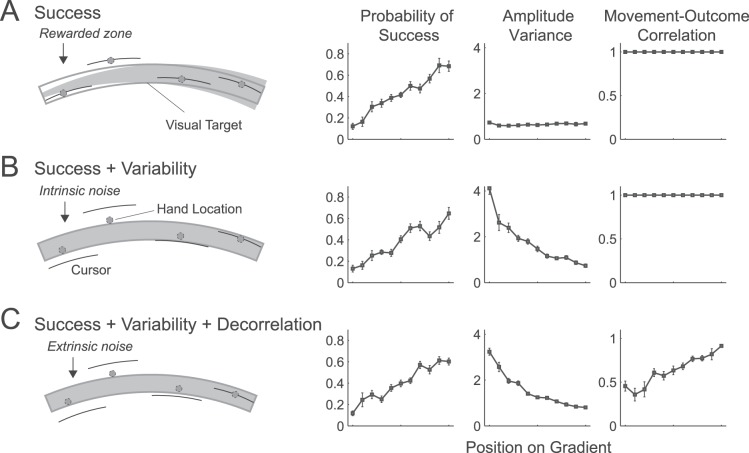
Manipulation of reward gradient in Experiment 1. (A) By decreasing the size of the rewarded zone along the 10° reward gradient, we decreased the probability of scoring a point on one side. The visual target remained the same. Cursor feedback was veridical, thus variability and correlation were constant along the gradient. (B) By exaggerating the extent error, we increased variability and reduced the probability of task success. For each position along the gradient, a tight correlation between movement and outcome was preserved. (C) By adding extrinsic noise to the amplitude feedback, we reduced task success, increased variability and reduced the correlation between action and outcome.

We controlled the available feedback signals via three independent manipulations (Fig. 3). In the success condition, we manipulated the width of the region in which the cursor had to terminate for a point to be scored. This manipulation increased the probability of task success in one direction, while leaving the variability of outcome (the visually indicated reach amplitude) unchanged. Note that the size of the rewarded zone was not explicitly indicated to participants and the actual target had the same width along the whole extent of the arc. This led to a number of trials in which the cursor either stopped outside the target and participants still received a point or inside the target and participants didn’t receive a point. However, none of the participants commented on this incongruence in the feedback. This was most likely because the cursor continued to move with the hand even after the computer program had detected the end of the trial, allowing participants to ascribe any discrepancy to small corrective movements after a trial end. The size of the rewarded zone was scaled such that for the average movement variability calculated across a representative sample of participants the chance of scoring a point would have been 0.2 one side of the gradient, and 0.8 on the other side. Across all the participants, these probabilities were approximately achieved (see Fig. 3).

In the success+variability condition, we magnified the reach amplitude error by a scaling factor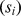. This was achieved by presenting the endpoint feedback (*c*) not at the actual hand position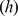, but slightly further away from the target (*t*).
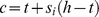



The magnitude of the scaling factor for intrinsic noise
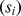
was determined by the region on the gradient they reached. For example, if participants overshot the target by 1 cm, at a region on the gradient where the scaling factor is 1.5, the cursor would be presented 1.5 cm past the target. The scaling factor was again chosen such that the average participant would have a probability of success of 0.2 on one side of the gradient, and 0.8 on the other side. In contrast to the success condition, however, this manipulation also increased the variability of participants’ movement extent.

In the success+variability+decorrelation condition, we added extrinsic variability in form of a Gaussian-distributed random variable to the feedback of the cursor extent on each trial.
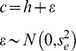



The magnitude of the extrinsic noise
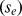
was also determined by the gradient region
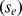
and was calibrated such that the outcome variability observed on the screen, and hence the probability of the task success, was equivalent to the previous condition. However, in contrast to the intrinsic noise condition, extrinsic noise also reduced the correlation between movement and visual outcome.

**Figure 4:**
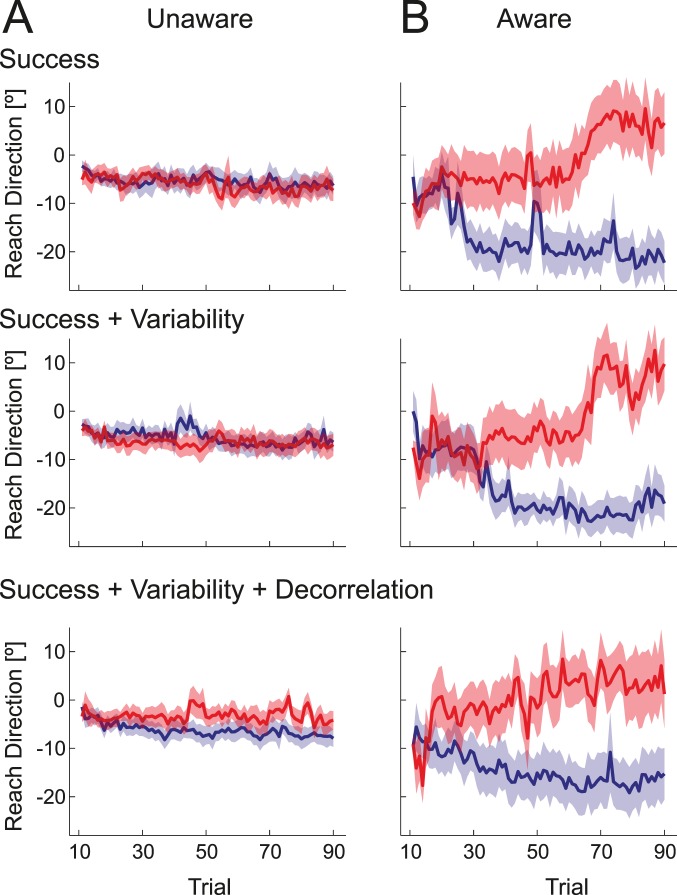
Manipulation of the gradient in Experiment 2. Data averaged over all participants. (A) Extrinsic noise added to the cursor feedback (movement amplitude) reduced success, increased variability, and reduced action-outcome correlation. (B) By adding extrinsic noise and simultaneously increasing the width of the rewarded zone, we increased outcome variability, but kept the probability of success stable across the gradient. (C) By increasing intrinsic noise (scaling of extent error) in one direction and extrinsic noise in the other direction, we varied the action-outcome correlation, but left probability of success and outcome variability stable across the gradient. The illustration of this condition shows the variance of amplitude error to be similar at all reach locations but on the far left the noise is extrinsic whereas to the right it becomes progressively more intrinsic.

In Experiment 2 (Fig. 4), we asked whether variability or action-outcome correlation might induce motor learning in the absence of variations in overall task success. We first replicated the third condition from Experiment 1, in which extrinsic noise was added so that task success, variability, and action-outcome correlation all varied along a gradient. In the variability+decorrelation condition, we magnified the extent error in one direction, and increased the size of the rewarded zone in the same direction (Fig. 4b). Consequently, the expected probability of task success should be 0.5 for all regions of the gradient, where one side of the gradient offered lower variability and higher action-outcome correlation. In the decorrelation condition, we applied a gradient that increased intrinsic noise in one direction and extrinsic noise in the opposite direction (Fig. 4c). This led to a higher movement-outcome correlation on one side of the gradient, while keeping the probability of task success and the total variability constant for all regions.

### Participants

Twenty-three healthy, right-handed participants (eight females, mean age 23.6) took part in Experiment 1. Twenty-four healthy right-handed participants (eleven females, mean age 24.5) took part in Experiment 2. One participant in Experiment 2 reported adopting the deliberate strategy of ignoring the visual feedback on the screen, and partly closed his eyes during the experiment. We therefore excluded this data set from further analysis. Written consent was obtained before the start of the experiment, and all procedures were approved by the ethics committee of the University College London.

### Apparatus and Stimuli (Technical Details)

The experimental setup was the same for both experiments. Participants were seated in front of a visual display with their foreheads positioned against a padded headrest. They made quick reaching movements with their right hands while holding a custom-built robotic device that recorded the position of the hand at 200Hz. Through a mirror above their hands, they viewed a display that was calibrated to provide visual feedback of the hand movement. The setup prevented participants from seeing their actual hand position at any time. After participants moved the cursor into the start position, an arced target spanning 90° with a radius 12 cm around a start position appeared (Fig. 2). To avoid online corrections, participants were instructed to make rapid movements towards the target, and we withdrew visual feedback during the movement. The movement started when the velocity threshold exceeded 3.5 cm/s and terminated when the velocity dropped below 3.5 cm/s for at least 40ms. At movement end, the cursor indicating the reach extent was displayed for 500ms. On rewarded trials the target and cursor turned red and participants observed an animated explosion of the target box. On unrewarded trials the target and cursor turned green. If the movement time limit of 700ms was exceeded, the target and cursor turned blue, and participants scored zero points for that trial. At the end of each trial, the manipulandum passively guided the hand back to the start position; cursor feedback was removed until the hand was within 3.5 cm of the start position.

The width of the rewarded zone (reach extents that would be rewarded) varied from 0.13 cm to 0.77 cm (0.44 to 2.22 Exp. 2), the scaling factor for internal noise from 1.17 to 5.29 (.38 to 1.9 Exp. 2) and the SD of the externally imposed error from 0.30 to 2.92, (0.05 to 3.23 Exp. 2). Each of these was calculated such that the probability of success would change linearly from 0.2 to 0.8, assuming that reach amplitudes were normally distributed with a SD of 0.55 cm. In a number of pilot experiments, subjects exhibited poor learning if the reward probability was varied gradually over the 90° target. We therefore varied the probability of success over a 10° region. To ensure that all participants experienced the gradient of feedback along the solution manifold the same way, we shifted the center of the gradient with the *current mean direction (m)* of the reach. This direction was calculated online as a low-pass filter of the reach direction (*y*) of the preceding trials:
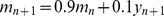



When the center of the gradient reached ±25°, it stopped moving with the participant’s behavior.

Participants experienced each of the three experimental conditions twice, once with the gradient biased to the left and once to the right. Participants were tested in a single session. To ensure that participants started out each block with a similar reach direction, each block started with ten trials that required participants to reach towards a square target presented at an offset of −7.5°. For these trials a veridical cursor was presented indicating both reach amplitude and direction. The particular angle was chosen because it was the mean preferred reach direction in a pilot study using the same task. This was followed by 80 trials of reaching to the redundant target, in which one of the three gradients was imposed in one direction. Each condition was one single uninterrupted block of 80 trials. Blocks with a flat gradient containing 40 trials were interleaved between test conditions to washout the effect of the previous block. The directional bias of the gradients always alternated (left/right) and conditions were counterbalanced pseudo-randomly.

After the experiment, participants were interviewed to determine whether they had become aware that varying the reach direction was an important dimension to better control reach extent. We first let them report freely any strategy that they may have used during the task to improve their performance. We then told them that there had been a hidden dimension that had influenced the task success, and instructed them to guess which dimension this was. Finally, we told them that success varied with the direction of the reach and asked them to guess whether in the last gradient block the better side of the target was on the left or right side. Participants who mentioned during the free report that they thought that movement direction was critical were classified as aware.

To determine whether awareness played a causal role in learning, we also measured learning in Experiment 1 for an additional five participants who were explicitly instructed before the experiment began. These participants were given the same task instructions as other participants and were informed that task success was dependent on producing the correct reach amplitude; however, they were also instructed that some reach directions may be easier than others.

### Data Analysis

As the main variable of interest, we determined the amplitude and direction of the primary movement. These were determined as the position of the hand after the movement end was detected (hand velocity <3.5 cm/s for 40ms).

To assess learning, we contrasted the two blocks for each participant under the same condition when the gradient was oriented to the left and right. For each block, we calculated the average reach direction for the last 40 trials. Learning was then measured as 50% of the angle between the two blocks, i.e., the average angular change in the direction of the imposed gradient. To test whether learning was significant, we used a one-tailed t-test on whether this learning score was bigger than zero. All other tests of groups and comparisons between conditions were two-sided.

We assumed that the direction of exploration would on average be uncorrelated across trials. We therefore used Gaussian process regression [25] to separate the overall variability into a slowly drifting component (resulting from gradual learning and accumulated noise along the solution manifold [26]) and a component that is independent across trials (consisting of output motor noise and exploration). Specifically, we modeled the co-variance between the reach direction (*y*) of trial *n* and *m* as:
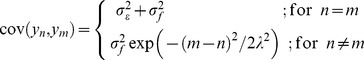



The hyperparameters, i.e., the variance of the random component (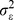), and variance (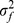) and length scale (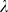) of the drifting component, were estimated by maximizing the likelihood of the data under the full model using the Matlab function *minimize* [25]. These fits were performed individually for each block of trials. We then used the standard deviation of the random process (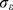) as a proxy for the amount of exploration.

## Results

### Experiment 1

Our first question was whether the arbitrary manipulation of success feedback could induce learning along a solution manifold. Izawa et al. [18] found good learning in a similar task. However, in that task, participants were explicitly aware that reach direction determined task success. Here we used a design in which task success depended primarily on an instructed dimension (reach amplitude), but in which another dimension (reach direction) provided secondary modulation. Hence, the first 18 participants were not explicitly made aware of the dimension they needed to change in order to improve performance.

**Figure 5:**
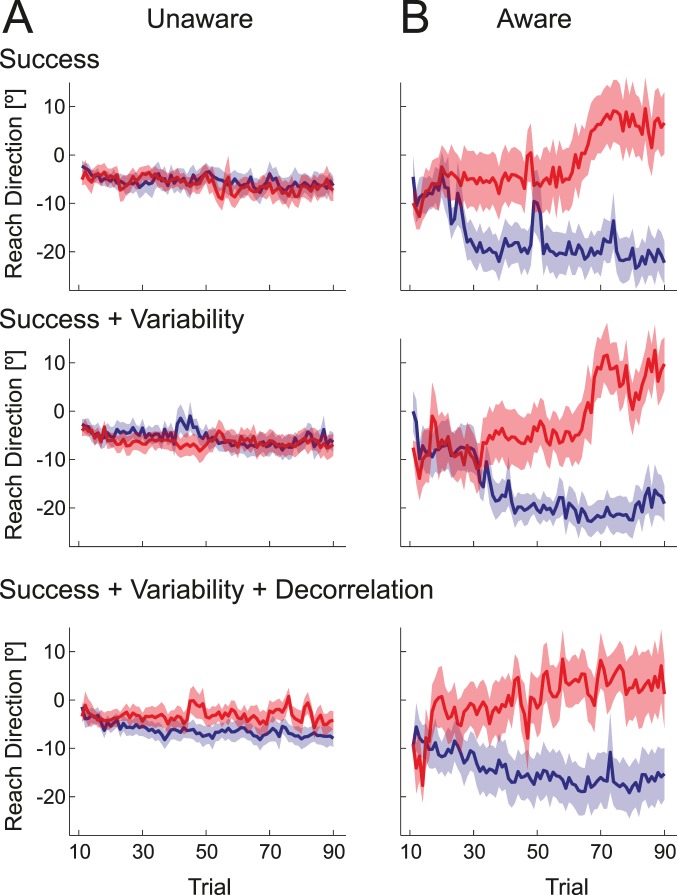
Average actual movement angle (deg) for blocks with a gradient to the left (blue) or right (red) in Experiment 1. (A) Participants who were not aware that movement direction mattered to the task showed minimal learning. Significant learning was only observed in a condition in which success, variability, and decorrelation all indicated the to-be-learned movement direction. The first ten movements were excluded, as movement direction here was dictated by an explicit target. (B) Participants who were aware that movement direction was critical to task success showed good learning. Significant learning was observed in all three conditions. See Figure S1 for individual traces.

**Figure 6:**
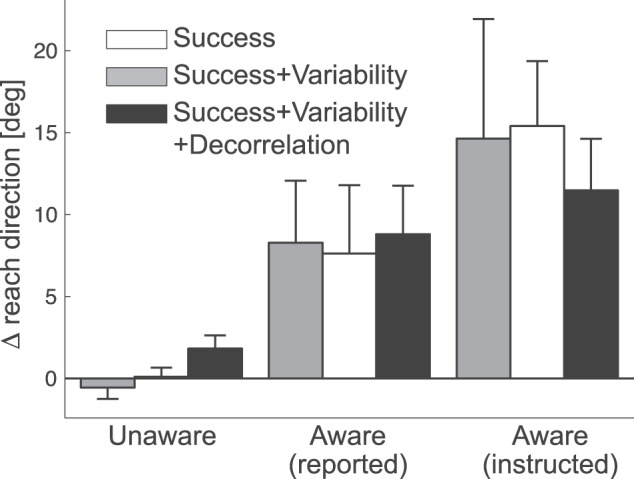
Average change in reach-direction in direction of the gradient for Experiment 1. The three left bars indicate the average change of reach direction (°) for the N = 13 participants who did not reported awareness of the critical dimension during debriefing. The middle three right bars indicate data from N = 5 participants who reported awareness. The right three bars are N = 5 participants who were informed of the critical dimension at the beginning of the experiment. Error bars indicate between-person standard error of the mean.

In the post-interview, 13 of the 18 participants reported no explicit awareness that movement direction mattered for the probability of task success. These participants were genuinely surprised that the direction (despite instructions) mattered. Even when asked to guess the direction that was better in the last block, only 7 out of 13 participants guessed correctly, a number not significantly different from chance, *p*  = 0.29. Overall, this group showed very little learning (Fig. 5a, Fig. 6). Conversely, the remaining 5 of the 18 participants reported that they had become aware of the critical dimension. The average learning curves for this group can be seen in Fig. 5b. These participants clearly adjusted the movement angle in the direction of the gradient in all conditions (Fig. 6, middle bars). Individual learning curves are shown in Figure S1.

Averaging across all conditions, the aware participants changed their reach in the direction of the gradient (8.23° +−2.70°) much more than the unaware participants (0.45° +−0.47°, *t*_(16)_  = 4.398, *p*<0.001). Furthermore, no significant difference was found in learning between the 7 unaware participants who guessed the last direction correctly and those 6 who guessed incorrectly, *t*_(11)_  = 0.631, *p*  = 0.541.

While these results may indicate that awareness leads to better learning, it is equally possible that better learning ability leads to an increase in the probability of becoming aware. To explicitly test whether awareness can play a causal role in the better learning, we ran five additional participants who were instructed at the beginning of the task that the direction of movement may matter (see methods). These participants all showed a large change in the direction of improved task success (13.8° +−3.40°, see Fig. 6, right bars). Although the learning was slightly larger than that observed for the group that became aware during the course of the experiment, this difference was not statistically significant, *F*_(1,8)_  = 1.668, *p*  = 0.232. For further analyses we therefore combined the two groups, if not otherwise stated. When asked to state the direction of the gradient in the last block, all instructed participants and 4 out of 5 of the participants who became aware reported the correct direction.

One possible reason for the improved learning in the aware participants is that they may have explored more along the solution manifold. Larger exploratory variability would indeed increase the amount of information available regarding the gradients that we employed. To quantify this observation, we decomposed the time series into a component that captures the slow drift across the block (i.e. learning), and a component that captures the trial-by-trial variability around this drift (see methods). Assuming that the direction of exploration would be on average uncorrelated across trials, we used the SD of the random trial-by-trial component as a proxy for exploration. Indeed, we found that the aware participants had a significantly higher standard deviation than did the unaware participants, *t*_(21)_  = 2.974, *p*  = 0.007.

**Figure 7:**
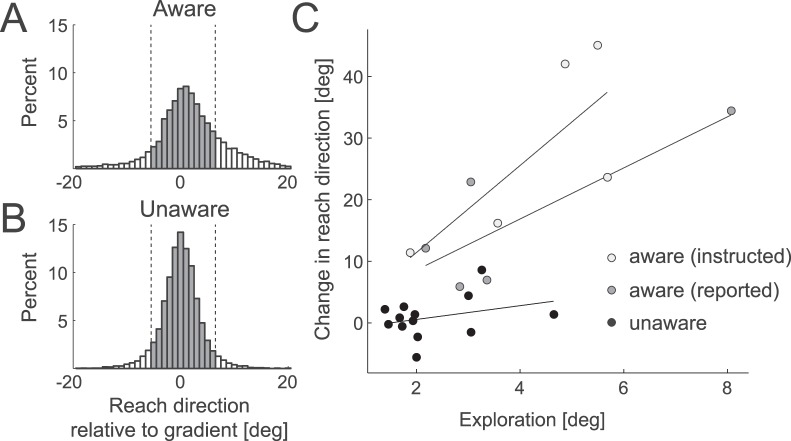
Role of exploration in learning in Experiment 1. (A) Histogram of the distribution of endpoint angles relative to the current position of the gradient. Positive angles indicate the direction of increased success probability. The gradient – the area over which the task success probability changed, is highlighted in gray (10° around the current mean). Aware participants showed substantial exploration; the bias to terminate movement on the rewarded side of the gradient arises from the fact that the gradient stopped moving with the average reach direction at ±25°. (B) Unaware participants explored less, but still experienced the full gradient. (C) Relationship of exploration, as measured by the uncorrelated component of reach direction variability, and learning for aware (white: instructed, gray: reported) and unaware (black) participants, with regression lines plotted for respective groups.

Can the difference in exploration fully account for the differences found in learning? There are some reasons to doubt this possibility. First, the reward gradient moved with each participant’s current mean and was relatively steep, such that even unaware participants sampled the whole gradient (Fig. 7a,b). Furthermore, when plotting the amount of exploration against the amount of learning (Fig. 7c), one can see that the aware and unaware groups overlapped considerably in terms of the amount of exploration, but still appeared to differ in the amount of learning. When removing the linear effect of the increased exploration using an ANCOVA, the difference in learning between the aware and unaware group remained significant, *F*_(1,20)_  = 17.631, *p*<.0001.

In sum, our results underline the critical importance of conscious awareness of the dimension in motor space that needs to change to increase the probability of success. While an increase in exploration along the critical movement dimension appears to be one mechanism through which awareness can increase learning speed, it is likely that it also changed the way participants learned from task rewards.

The second aim of the study was to determine whether people can learn from rewards without awareness of the critical movement dimension, and how different learning signals, all of which could indicate movement outcome quality, might differ in their ability to drive learning. The aware participants (Fig. 6) learned equally well in all three conditions, *F*_(2,18)_  = 0.085, *p*  = 0.918. Importantly they also learned significantly from task success alone, *t*_(9)_  = 2.853, *p*  = 0.009, replicating previous results [18].

In contrast, we found significant differences between the three task conditions in the unaware group, *F*_(2, 24)_  = 3.879, *p*  = 0.035. We observed no learning from task success alone, *t*_(12)_ = −0.816, *p*  = 0.785, nor when the gradient indicated both success and variability, *t*_(12)_  = 0.190, *p*  = 0.426. Only when success, variability and action-outcome correlation all varied in the same direction along the gradient, did participants learn significantly, *t*_(12)_  = 2.230, *p*  = 0.023, albeit to a significantly lesser extent than did aware participants.

These differences occurred despite the fact that the gradient of reward probability appeared to be well matched across the three conditions (Fig. 3). To quantify possible differences in the reward gradient, we submitted the proportion of trials where a reward was obtained to an ANOVA with the factors gradient zone (1–11) and task condition. Because the integrity of the reward gradient should not depend on awareness, the ANOVA was conducted on all participants. There was no significant difference between task conditions in terms of overall reward, *F*_(2,44)_  = 2.831, *p*  = 0.07, and there was no significant interaction between the gradient zone and condition, *F*_(20,440)_  = 1.039, *p*  = 0.414. On average, the gradient for the success+variability+decorrelation condition was even slightly shallower than in the other conditions (see Fig. 3), thereby strengthening our claim that the increased learning in this condition cannot be attributed to a clearer feedback gradient.

Finally, we asked whether the difference between the task conditions could be due to a difference in the amount of exploration. For the unaware participants we found no significant difference between the task conditions, *F*_(2,24)_  = 0.32, *p*  = 0.726. Even when accounting for differences in exploration using an analysis of covariance (ANCOVA), the differences in learning remained significant, *F*_(2,24)_  = 3.933, *p*  = 0.033. Thus, exploration differences cannot account for the learning differences observed across task conditions.

### Experiment 2

Experiment 1 provided evidence that conscious awareness of the dimension one must explore dramatically improves learning from rewards. In the absence of conscious awareness we found learning only when task success, variability of the outcome variable, and action-outcome correlation all indicated the better solution along the solution manifold (here movement direction). This configuration of signals occurs when extrinsic or uncontrollable noise is higher for one location along the solution manifold than for another. In a second experiment we explored whether variability and action-outcome decorrelation alone in the absence of any gradient in the probability of the explicit reward could drive learning.

By manipulating the width of the target, and the amount of extrinsic and intrinsic noise separately, we created three different conditions, each of which had different combinations of the three possible learning signals (Fig. 4). The first condition, by adding just extrinsic noise, contained all three signals, repeating the success+variability+decorrelation condition from Experiment 1. The second condition had the same gradient for variability and decorrelation, but not for average task success, which was constant across the whole gradient. The third condition tested the influence of movement-outcome decorrelation alone. In this case, both the probability of success and the variability were constant across movement directions.

**Figure 8:**
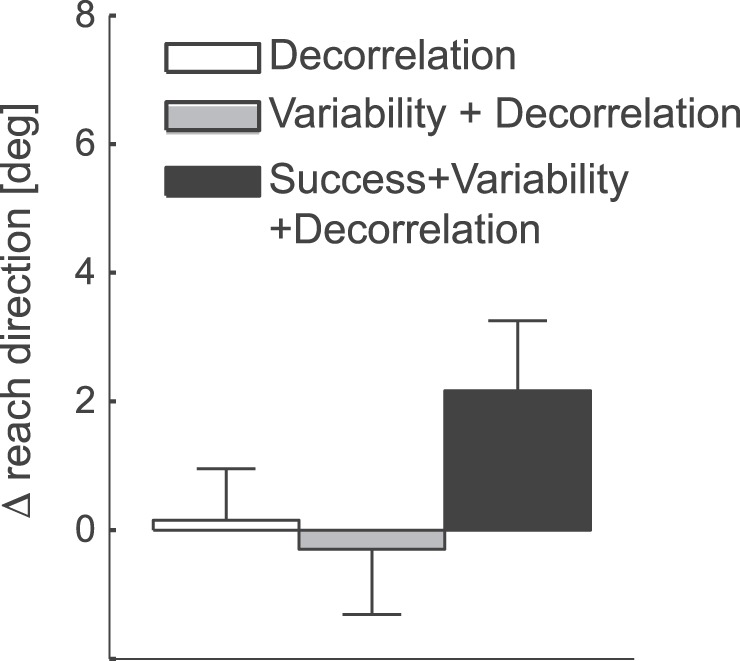
Average change in reach-direction in Experiment 2. Positive values indicate a shift in reach direction to the side of the target that was associated with better control of the reach extent. Only data from unaware participants (N = 21) is shown. Error bars indicate between-person standard error of the mean.

In Experiment 2, only two of the 23 participants became aware that reach direction was a critical factor. This is most likely due to the fact that the explicit reward only varied in one of the three conditions. Here we consider only the 21 unaware participants (see Fig. 8). We found that neither the variance+decorrelation condition, *t*_(20)_ = −0.294, *p*  = 0.614, nor decorrelation alone condition, *t*_(20)_  = 0.191, *p*  = 0.425, generated significant learning. However, in the success+variability+decorrelation condition we replicated the results of Experiment 1, finding significant learning, *t*_(20)_  = 1.996, *p*  = 0.030. Again, the exploratory SD of the movements was not significantly different between the conditions (*F*_(2,40)_  = 0.244, *p*  = 0.784). Thus, the difference in learning between the conditions was not caused by differences in exploration.

## Discussion

In this study we asked how motor performance improves in multi-dimensional, redundant, control problems. We considered whether task success, signaled by external monetary reward, would be a sufficient signal to improve motor output along an unknown dimension. Previous studies [17], [18], which involved variants of the task used here, clearly showed robust learning from task success alone. However, in both of those studies, the participants knew that success would vary with movement direction. In our task, participants were not given this information explicitly, and they were therefore uncertain as to which of the many movement parameters (movement speed, arm posture, initial acceleration, curvature, grip strength, etc.) they had to vary in order to control the task-relevant variable of movement extent more proficiently and to produce rewarded outcomes. In this respect, our task reflects the incidental nature of many natural motor-learning tasks, such as improving one’s game of darts in the absence of coaching. Of course, the redundant dimension in our task was itself somewhat artificial. For future studies, higher dimensional tasks may afford greater opportunity to explore more ethologically valid forms of redundancy [27]–[30].

Our experiments clearly showed that awareness of the critical dimension during motor learning is the key factor which allows learning from arbitrary rewards: only participants who were instructed or became aware that movement direction was the critical variable, changed their behavior in the success-only condition. In contrast, participants who did not become aware did not show learning from explicit rewards alone.

Awareness could have influenced learning through at least two mechanisms: First, it may have increased exploration along the relevant dimension. Exploration is a key component of the process by which reinforcement learning leads to improved outcomes [14], [31]. We measured exploration directly using the uncorrelated output variability after subtracting slowly varying trends from the data. This is how exploration should appear in our task, even though under a normative treatment, exploration involves deterministic choices [32]. Indeed, in our task, awareness was associated with increased output variability along the solution manifold. This increased exploration also appeared to be connected to better learning (Fig. 7c). Nevertheless, after removal of the influence of the higher exploration on learning using a linear (ANCOVA) model, the difference between aware and unaware participants remained significant. This suggests that increased exploration may not have been the only mechanism by which awareness promoted motor learning in this task. An important caveat is, however, that this analysis rests on a linear model, whereas the underlying relationship between exploration and learning may be non-linear.

As a second possible mechanism, awareness may have been used to bias the way in which the reinforcement signal was employed for learning. One of the core problems for reinforcement learning is the use of a scalar reward signal to learn in a high-dimension space e.g., [33]. This so-called ‘structural credit assignment’ problem has long been recognized in the field of conditioning [34]–[36] and perceptual learning [37], [38], where it is solved by an attentional mechanism akin to boosting the speed of learning (formally, the learning rate) for just the parameters deemed important for behavioral change. Unaware participants who tried exploring the movement direction dimension might have failed to allocate learning to it. Indeed, in the post-task interview, they reported paying attention to many other parameters, including movement speed, arm posture, and grip configuration. Whether through single or joint effects of these possible mechanisms, our findings emphasize that awareness is an important and underappreciated aspect of reward-based motor skill learning [39].

The importance of awareness in finding the optimal region on the solution manifold based on rewards contrast starkly with the automaticity of error-based adaption, which keeps the system on the manifold. Adaptation to perturbation occurs implicitly and without the need for conscious awareness. Indeed, adaptation is present even when it conflicts with explicit cognitive strategies [8], when perturbations are not relevant to a task [40]or when people are informed that the perturbations are random and such that adaptation would not improve performance [11], [41].

Unaware participants did not learn from task success feedback alone. Instead, they only showed significant learning when the best movement direction was also characterized by two additional second-order statistics of the movement outcome. The first of these higher-order signals was the variability of the motor outcome, consistent with a central tenet of many current theories of motor control, which state that the nervous system chooses solutions that reduce variability [12], [19]–[22], [42]. While reducing variability is usually associated with improved task success, in our task we manipulated variability and success independently. The results suggest that the motor system is sensitive to variability alone, and should therefore have a way of assessing variability independent of task success, perhaps by accumulating statistics such as the absolute or the squared prediction error.

Interestingly, however, output variability was not the only second-order statistic to which the motor system was sensitive. When we increased output variability by magnifying natural errors, no learning occurred. Only when output variability came from the imposition of random external noise, did participants shift their motor behavior in the reinforced direction. This was true even though participants experienced the same gradient in terms of total output variability and task success in both conditions. What distinguished the two conditions was that during the magnification of internal noise, the correlation of the physical movement and the visual outcome was preserved, whereas the addition of external noise degraded this relationship. Our results therefore suggest that the motor system is sensitive to this variable and prefers solutions in which the outcome can be predicted well from the movement.

Why should the nervous system take into account outcome variability and action-outcome correlation *independent of task success*? While, by definition, overall task success is all that matters for a given task, optimizing these two second-order statistics may enable the motor system to retain good performance when the environmental circumstances change. For example, solutions with high variability may be sufficient for tasks that have a very lenient success criterion, but it may be preferable to arrive at solutions with lower variability in case task requirements become stricter. Furthermore, high movement-outcome correlations, which indicate high controllability, provide the system with the opportunity to react quickly to changes in task goals or dynamics. They also indicate that reductions in exploratory noise would reduce the output variability if necessary.

Clearly, however, the unaware participants’ learning was relatively ineffective. This agrees with the general sloth of skill acquisition, particularly when compared with first-order error based learning, which can lead to improvements after a few movements. Indeed, it is possible that our results arose because of the integration of three relatively weak signals, rather than to the fact that any of them was strictly necessary to drive learning in the absence of awareness. Whether learning can be induced with any signal alone is an important question for further studies, for instance by exposing the participants to much longer training episodes to obtain reliable learning results. Furthermore, we were not able to determine whether the additional signals provided direct information for learning, information about the relevant dimension that governed success, or both.

While our results show that learning without awareness requires the confluence of multiple learning signals, they do not speak to the actual learning mechanisms involved. As discussed by Huang et al. [43], conventional, discrete, reinforcement learning literature would offer model-based and model-free control methods [44]. Model-free methods would be driven by a scalar measure of task success. By contrast, model-based methods would learn the mapping from actions to outcome (here, various moments of the statistics of performance) and invert that model to work out what to do. The signature of model-based learning is flexibility, i.e. rapid adaptation when circumstances change.

An important example for such learning is provided by the “reaching under risk” studies [45], in which participants aim at different spatial configurations of reward and penalty zones. The studies show that participants can use knowledge about their own variability to make optimal choices [46], [47], see also [48], [49], and that they can learn, to a certain degree at least, a new structure of variability [50], [51]. The critical difference between our study and the reaching under risk paradigm is that in the latter, variability and change in behaviour both occur in the same task-relevant dimension. In many real-world tasks (for example dart throwing), reductions in end-point variability can be achieved by changes in different dimensions, for example the way one angles the elbow while throwing. Thus, a change that leads to a different degree of variability does not necessarily change the average endpoint of the movement in the task-relevant plane. It is this situation that our current study addresses.

Here we have looked at how reward signals influence motor learning over the course of 80 trials. At a different timescale, other studies have shown that the average reward obtained during a training session influences the consolidation of the memory trace [52]. In that report the authors argued that rewards did not serve as an informative signal that indicated which of several movements was more successful (i.e., rewards did not change within-session learning) but rather served as a signal to help consolidate the entire training session. Converging evidence for a more tonic (lower frequency) influence of reward signals in motor learning comes from a series of studies on the role of dopaminergic projections from the ventral tegmental area (VTA) to primary motor cortex in the rat [53]. The elimination of these connections leads to severe deficits in learning a pellet-retrieval task [54], and the learning in the lesioned animals can be recovered through administration of levodopa [55]. While such pharmacological intervention can raise tonic levels of dopamine in the motor system, it is unlikely that it could reinstate the phasic signals that appear to mark the success of particular actions. Thus, it is possible that higher-order signals (i.e., the variability of prediction errors) and reinforcement signals may contribute to motor learning on different time scales.

In summary, our experiments show the critical importance of attention or awareness of the critical movement dimension in a multi-dimensional control task to utilize reward signals for motor learning. In absence of such clear guidance for exploration and credit assignment, learning was only present when higher-order signals, including the outcome variability and action-outcome decorrelation, were aligned with extrinsic reward signals. Even then, learning was much slower than in cases where awareness was present. This finding is congruent with a number of failed attempts from our (O’Sullivan & Diedrichsen, unpublished results) and other (Koerding & Wolpert, personal communication) laboratories to obtain compelling and robust reinforcement learning in higher-dimensional control tasks. It may also help explain why skill learning is laborious, with substantial improvements often only being achievable through the directive influence of a coaching program.

## Supporting Information

Figure S1 **Individual data from Experiment 1 for change in movement end angle (deg) for blocks with a gradient to the left (blue) or right (red).** Each line represents data from an individual participant/block. Data is shown for the 3 conditions (success only, success+variability, success+variability+decorrelation) and for 3 groups of participants (unaware, aware, and instructed). For the first 10 trials an explicit target was presented at −7.5°.
(EPS)

## References

[1] Debicki DB, Gribble PL, Watts S, Hore J (2004) Kinematics of wrist joint flexion in overarm throws made by skilled subjects. Exp Brain Res 154: 382–394. 14598003 10.1007/s00221-003-1673-4

[2] Hore J, Watts S (2005) Timing finger opening in overarm throwing based on a spatial representation of hand path. J Neurophysiol 93: 3189–3199. 15911892 10.1152/jn.01268.2004

[3] Hore J, Watts S, Martin J, Miller B (1995) Timing of finger opening and ball release in fast and accurate overarm throws. Exp Brain Res 103: 277–286. 7789435 10.1007/BF00231714

[4] Latash ML, Scholz JP, Schoner G (2002) Motor control strategies revealed in the structure of motor variability. Exerc Sport Sci Rev 30: 26–31. 11800496 10.1097/00003677-200201000-00006

[5] Scholz JP, Schoner G (1999) The uncontrolled manifold concept: identifying control variables for a functional task. Exp Brain Res 126: 289–306. 10382616 10.1007/s002210050738

[6] Wolpert DM, Diedrichsen J, Flanagan JR (2011) Principles of sensorimotor learning. Nat Rev Neurosci 12: 739–751. 22033537 10.1038/nrn3112

[7] Tseng YW, Diedrichsen J, Krakauer JW, Shadmehr R, Bastian AJ (2007) Sensory prediction errors drive cerebellum-dependent adaptation of reaching. J Neurophysiol 98: 54–62. 17507504 10.1152/jn.00266.2007

[8] Mazzoni P, Krakauer JW (2006) An implicit plan overrides an explicit strategy during visuomotor adaptation. J Neurosci 26: 3642–3645. 16597717 10.1523/JNEUROSCI.5317-05.2006 PMC6674132

[9] Shadmehr R, Mussa-Ivaldi FA (1994) Adaptive representation of dynamics during learning of a motor task. J Neurosci 14: 3208–3224. 8182467 10.1523/JNEUROSCI.14-05-03208.1994 PMC6577492

[10] Thoroughman KA, Shadmehr R (2000) Learning of action through adaptive combination of motor primitives. Nature 407: 742–747. 11048720 10.1038/35037588 PMC2556237

[11] Diedrichsen J, Hashambhoy YL, Rane T, Shadmehr R (2005) Neural correlates of reach errors. Journal of Neuroscience 25: 9919–9931. 16251440 10.1523/JNEUROSCI.1874-05.2005 PMC1479774

[12] Sternad D, Abe MO, Hu X, Muller H (2011) Neuromotor noise, error tolerance and velocity-dependent costs in skilled performance. PLoS Comput Biol 7: e1002159. 21966262 10.1371/journal.pcbi.1002159 PMC3178634

[13] Muller H, Sternad D (2009) Motor learning: changes in the structure of variability in a redundant task. Adv Exp Med Biol 629: 439–456. 19227514 10.1007/978-0-387-77064-2_23 PMC3776417

[14] Sutton RS, Barto AG (1998) Reinforcement learning. Cambridge, Massachusetts: MIT Press

[15] Peters J, Schaal S. Using reward-weighted regression for reinforcement learning of task space control; 2007; Honolulu, Hawaii. 262–267.

[16] Peters J, Schaal S (2008) Reinforcement learning of motor skills with policy gradients. Neural Netw 21: 682–697. 18482830 10.1016/j.neunet.2008.02.003

[17] Dam G, Kording K (2009) Exploration and exploitation during sequential search. Cogn Sci 33: 530–541. 21585479 10.1111/j.1551-6709.2009.01021.x PMC3403695

[18] Izawa J, Shadmehr R (2011) Learning from sensory and reward prediction errors during motor adaptation. PLoS Comput Biol 7: e1002012. 21423711 10.1371/journal.pcbi.1002012 PMC3053313

[19] Harris CM, Wolpert DM (1998) Signal-dependent noise determines motor planning. Nature 394: 780–784. 9723616 10.1038/29528

[20] van Beers RJ, Baraduc P, Wolpert DM (2002) Role of uncertainty in sensorimotor control. Philos Trans R Soc Lond B Biol Sci 357: 1137–1145. 12217180 10.1098/rstb.2002.1101 PMC1693018

[21] Todorov E, Jordan MI (2002) Optimal feedback control as a theory of motor coordination. Nat Neurosci 5: 1226–1235. 12404008 10.1038/nn963

[22] Todorov E (2002) Cosine tuning minimizes motor errors. Neural Comput 14: 1233–1260. 12020444 10.1162/089976602753712918

[23] Churchland MM, Afshar A, Shenoy KV (2006) A central source of movement variability. Neuron 52: 1085–1096. 17178410 10.1016/j.neuron.2006.10.034 PMC1941679

[24] Diedrichsen J, White O, Newman D, Lally N (2010) Use-dependent and error-based learning of motor behaviors. J Neurosci 30: 5159–5166. 20392938 10.1523/JNEUROSCI.5406-09.2010 PMC6632748

[25] Rasmussen CE, Williams CKI (2006) Gaussian processes for machine learning. Cambridge, Massachusetts: The MIT Press.

[26] van Beers RJ, Brenner E, Smeets JB (2013) Random walk of motor planning in task-irrelevant dimensions. J Neurophysiol 109: 969–977. 23175799 10.1152/jn.00706.2012

[27] Vetter P, Flash T, Wolpert DM (2002) Planning movements in a simple redundant task. Curr Biol 12: 488–491. 11909535 10.1016/s0960-9822(02)00715-7

[28] Liu X, Mosier KM, Mussa-Ivaldi FA, Casadio M, Scheidt RA (2011) Reorganization of finger coordination patterns during adaptation to rotation and scaling of a newly learned sensorimotor transformation. J Neurophysiol 105: 454–473. 20980541 10.1152/jn.00247.2010 PMC3023375

[29] Mosier KM, Scheidt RA, Acosta S, Mussa-Ivaldi FA (2005) Remapping hand movements in a novel geometrical environment. J Neurophysiol 94: 4362–4372. 16148276 10.1152/jn.00380.2005

[30] Mussa-Ivaldi FA, Casadio M, Danziger ZC, Mosier KM, Scheidt RA (2011) Sensory motor remapping of space in human-machine interfaces. Prog Brain Res 191: 45–64. 21741543 10.1016/B978-0-444-53752-2.00014-X PMC3517730

[31] Kaelbling LP, Littman ML, Moore AW (1996) Reinforcement Learning: A Survey. Journal of Artificial Intelligence Research 4: 237–285.

[32] Gittins JC (1979) Bandit processes and dynamic allocation indices. Journal of the Royal Statistical Society, Series B: 148–177.

[33] Sutton RS (1996) Generalization in reinforcement learning: Successful examples using sparse coarse coding. Advances in neural information processing systems: 1038–1044.

[34] Dayan P, Kakade S, Montague PR (2000) Learning and selective attention. Nat Neurosci 3 Suppl: 1218–1223 10.1038/81504 11127841

[35] Pearce JM, Hall G (1980) A model for Pavlovian learning: variations in the effectiveness of conditioned but not of unconditioned stimuli. Psychol Rev 87: 532–552. 7443916

[36] Kruschke JK (2001) Towards a unified model of attention in associative learning. Journal of Mathematical Psychology 45: 812–863.

[37] Roelfsema PR, van Ooyen A (2005) Attention-gated reinforcement learning of internal representations for classification. Neural Comput 17: 2176–2214. 16105222 10.1162/0899766054615699

[38] Roelfsema PR, van Ooyen A, Watanabe T (2010) Perceptual learning rules based on reinforcers and attention. Trends Cogn Sci 14: 64–71. 20060771 10.1016/j.tics.2009.11.005 PMC2835467

[39] Stanley J, Krakauer JW (2013) Motor skill depends on knowledge of facts. Front Hum Neurosci 7: 503. 24009571 10.3389/fnhum.2013.00503 PMC3756281

[40] Schaefer SY, Shelly IL, Thoroughman KA (2012) Beside the point: motor adaptation without feedback-based error correction in task-irrelevant conditions. J Neurophysiol 107: 1247–1256. 22157120 10.1152/jn.00273.2011 PMC3289459

[41] Donchin O, Francis JT, Shadmehr R (2003) Quantifying generalization from trial-by-trial behavior of adaptive systems that learn with basis functions: theory and experiments in human motor control. J Neurosci 23: 9032–9045. 14534237 10.1523/JNEUROSCI.23-27-09032.2003 PMC6740843

[42] Diedrichsen J, Shadmehr R, Ivry RB (2010) The coordination of movement: optimal feedback control and beyond. Trends Cogn Sci 14: 31–39. 20005767 10.1016/j.tics.2009.11.004 PMC4350769

[43] Huang VS, Haith A, Mazzoni P, Krakauer JW (2011) Rethinking motor learning and savings in adaptation paradigms: model-free memory for successful actions combines with internal models. Neuron 70: 787–801. 21609832 10.1016/j.neuron.2011.04.012 PMC3134523

[44] Daw ND, Niv Y, Dayan P (2005) Uncertainty-based competition between prefrontal and dorsolateral striatal systems for behavioral control. Nat Neurosci 8: 1704–1711. 16286932 10.1038/nn1560

[45] Trommershauser J, Maloney LT, Landy MS (2003) Statistical decision theory and the selection of rapid, goal-directed movements. J Opt Soc Am A Opt Image Sci Vis 20: 1419–1433. 12868646 10.1364/josaa.20.001419

[46] Trommershauser J, Maloney LT, Landy MS (2008) Decision making, movement planning and statistical decision theory. Trends Cogn Sci 12: 291–297. 18614390 10.1016/j.tics.2008.04.010 PMC2678412

[47] Gepshtein S, Seydell A, Trommershauser J (2007) Optimality of human movement under natural variations of visual-motor uncertainty. J Vis 7: 13 11–18. 10.1167/7.5.13 18217853

[48] Hudson TE, Maloney LT, Landy MS (2008) Optimal compensation for temporal uncertainty in movement planning. PLoS Comput Biol 4: e1000130. 18654619 10.1371/journal.pcbi.1000130 PMC2442880

[49] Battaglia PW, Schrater PR (2007) Humans trade off viewing time and movement duration to improve visuomotor accuracy in a fast reaching task. J Neurosci 27: 6984–6994. 17596447 10.1523/JNEUROSCI.1309-07.2007 PMC6672223

[50] Landy MS, Trommershauser J, Daw ND (2012) Dynamic estimation of task-relevant variance in movement under risk. J Neurosci 32: 12702–12711. 22972994 10.1523/JNEUROSCI.6160-11.2012 PMC3477850

[51] Trommershauser J, Gepshtein S, Maloney LT, Landy MS, Banks MS (2005) Optimal compensation for changes in task-relevant movement variability. J Neurosci 25: 7169–7178. 16079399 10.1523/JNEUROSCI.1906-05.2005 PMC6725228

[52] Abe M, Schambra H, Wassermann EM, Luckenbaugh D, Schweighofer N, et al (2011) Reward Improves Long-Term Retention of a Motor Memory through Induction of Offline Memory Gains. Curr Biol 21: 557–562. 21419628 10.1016/j.cub.2011.02.030 PMC3075334

[53] Luft AR, Schwarz S (2009) Dopaminergic signals in primary motor cortex. Int J Dev Neurosci 27: 415–421. 19446627 10.1016/j.ijdevneu.2009.05.004

[54] Molina-Luna K, Pekanovic A, Rohrich S, Hertler B, Schubring-Giese M, et al (2009) Dopamine in motor cortex is necessary for skill learning and synaptic plasticity. PLoS One 4: e7082. 19759902 10.1371/journal.pone.0007082 PMC2738964

[55] Hosp JA, Pekanovic A, Rioult-Pedotti MS, Luft AR (2011) Dopaminergic projections from midbrain to primary motor cortex mediate motor skill learning. J Neurosci 31: 2481–2487. 21325515 10.1523/JNEUROSCI.5411-10.2011 PMC6623715

