# Correction: When Money Is Not Enough: Awareness, Success, and Variability in Motor Learning

**DOI:** 10.1371/journal.pone.0097058

**Published:** 2014-05-02

**Authors:** The PLOS ONE Staff

Figure 4 is incorrect. The authors have provided a corrected version here.

**Figure 4:**
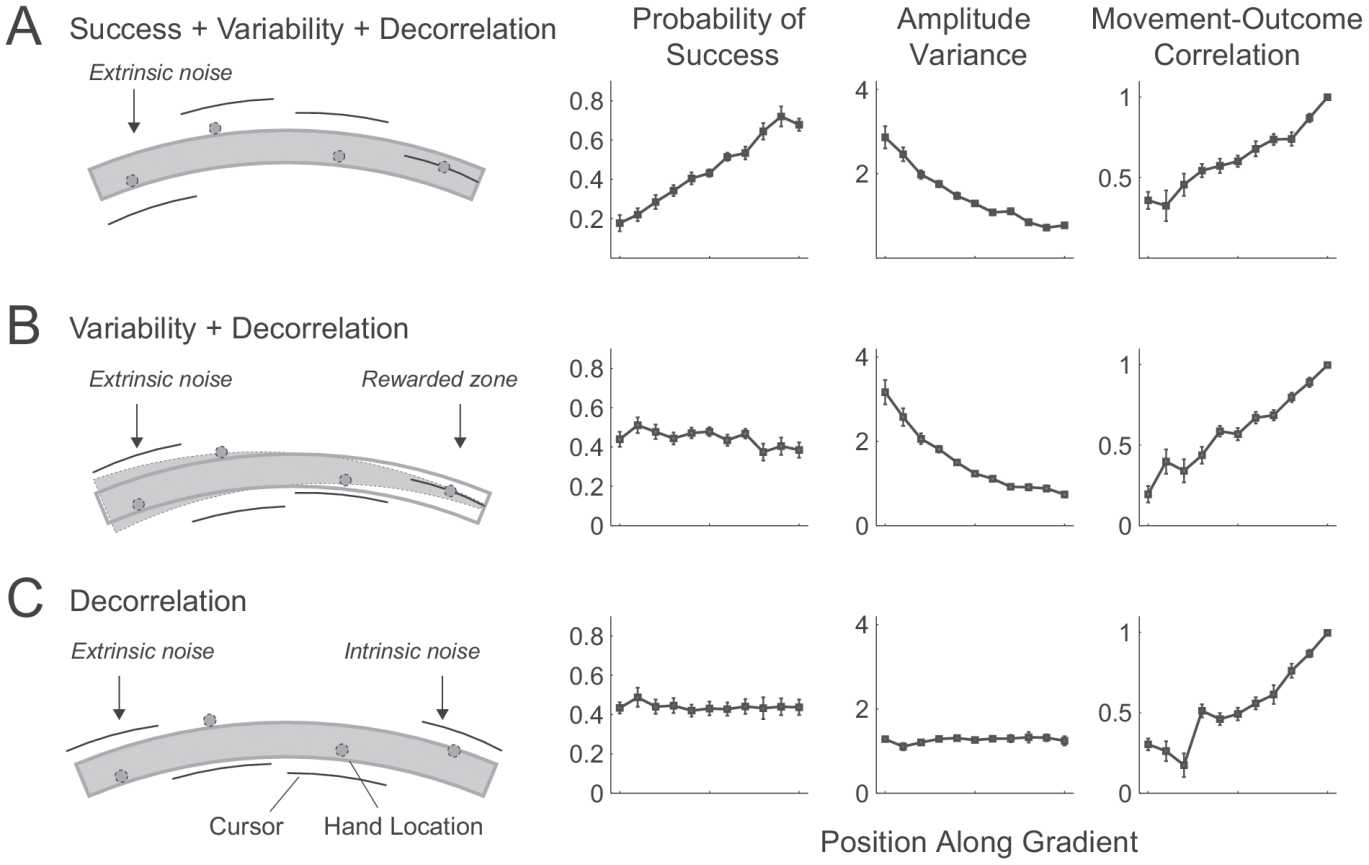
Manipulation of the gradient in Experiment 2. (A) Extrinsic noise added to the cursor feedback (movement amplitude) reduced success, increased variability, and reduced action-outcome correlation. (B) By adding extrinsic noise and simultaneously increasing the width of the rewarded zone, we increased outcome variability, but kept the probability of success stable across the gradient. (C) By increasing intrinsic noise (scaling of extent error) in one direction and extrinsic noise in the other direction, we varied the action-outcome correlation, but left probability of success and outcome variability stable across the gradient. The illustration of this condition shows the variance of amplitude error to be similar at all reach locations but on the far left the noise is extrinsic whereas to the right it becomes progressively more intrinsic.
